# Clinical significance of erythrocyte sedimentation rate-based stratification in a large retrospective SLE cohort

**DOI:** 10.3389/fimmu.2026.1767359

**Published:** 2026-03-27

**Authors:** Fan Wang, Yang Xu, Wei Zhang, Wenyou Pan, Lin Liu, Min Wu, Fuwan Ding, Huaixia Hu, Xiang Ding, Hua Wei, Yaohong Zou, Wei Kong, Yun Zhu, Xuebing Feng, Lingyun Sun

**Affiliations:** 1 Department of Rheumatology and Immunology, Nanjing Drum Tower Hospital, Affiliated Hospital of Medical School, Nanjing University, Nanjing, China; 2 Department of Rheumatology and Immunology, Nanjing Medical University Drum Tower Clinical Medical Hospital, Nanjing, China; 3 Department of Rheumatology, Huaian First People’s Hospital, Huaian, China; 4 Department of Rheumatology, Xuzhou Central Hospital, Xuzhou, China; 5 Department of Rheumatology, The Third Affiliated Hospital of Soochow University, Changzhou, China; 6 Department of Endocrinology, Yancheng Third People’s Hospital, Yancheng, China; 7 Department of Rheumatology, Lianyungang Second People’s Hospital, Lianyungang, China; 8 Department of Rheumatology, Lianyungang First People’s Hospital, Lianyungang, China; 9 Department of Rheumatology, Northern Jiangsu People’s Hospital, Yangzhou, China; 10 Department of Rheumatology, Wuxi People’s Hospital, Wuxi, China

**Keywords:** systemic lupus erythematosus, erythrocyte sedimentation rate, C-reactive protein, disease activity, infection

## Abstract

**Objectives:**

Erythrocyte sedimentation rate (ESR) is one of the most commonly used markers of inflammation in clinical practice. However, its value in predicting disease behavior in patients with systemic lupus erythematosus (SLE) remains controversial. The aim of this study was to determine the clinical significance of ESR in a large cohort of Chinese patients with SLE.

**Methods:**

Data of 1,217 patients with documented ESR values were extracted from a lupus database collected by Jiangsu Lupus Collaborative Group. The associations of ESR with diverse manifestations, disease activity, damage accruement, and concurrent infection status were evaluated using logistic regression, and receiver operating characteristic (ROC) curve analysis was performed to determine the optimal cutoffs for ESR and the C-reactive protein (CRP)/ESR ratio. The prognosis of patients with normal and high ESR was assessed using Kaplan–Meier analysis.

**Results:**

Of the patients in this cohort, 81.0% had increased ESR values. ESR elevation was independently associated with fever (OR = 1.664, 95%CI = 1.072–2.583), serositis (OR = 2.005, 95%CI = 1.105–3.64), interstitial pneumonia (OR = 3.394, 95%CI = 1.176–9.796), hypoalbuminemia (OR = 1.536, 95%CI = 1.076–2.193), and anemia (OR = 3.102, 95%CI = 2.213–4.348). Compared with the damage accrual score [the Systemic Lupus International Collaborating Clinics/American College of Rheumatology Damage Index (SDI)], there was a stronger correlation between the ESR value and the SLE Disease Activity Index (SLEDAI) score, and a value not less than 25 mm/h helped to define disease activity with high sensitivity. The increase of ESR was much slower than that of CRP at the time of infection, and a CRP/ESR ratio ≥0.5 had high specificity to depict the presence of infection. The 5- and 10-year survival rates of patients with elevated ESR values were worse than those with normal values.

**Conclusions:**

This study suggests that ESR is not only related to disease activity and prognosis but also to the patient’s autoimmune status. The CRP/ESR ratio helps distinguish the presence of infection in patients with SLE.

## Introduction

Systemic lupus erythematosus (SLE) is a prototypic autoimmune disease with a wide variety of signs and symptoms. During the disease process, elevated disease activity, newly occurred organ damage, and infection often co-occur and always pose a major challenge to clinical treatments (1–3). As the organ involvements in SLE are often inflammatory-related (4), it is important to monitor the inflammatory state of patients. Among the markers, erythrocyte sedimentation rate (ESR) has long been applied for the assessment of disease activity of systemic inflammatory diseases including SLE. However, due to the small number of samples in the majority of studies, the specific effect of an increased ESR on this disease remains controversial (5–9). In order to determine the role of ESR in SLE and discover the reasons leading to the discrepancies in previous studies, the data of Chinese patients with SLE retracted from the Jiangsu Lupus Database were analyzed here for their associations with clinical features, activity, infection, and outcomes.

## Patients and methods

### Data collection and definition

As previously reported, the medical records of over 2,500 hospitalized SLE cases have been collected by Jiangsu Lupus Collaborative Group since 2010 (10, 11). All patients fulfilled at least four of the revised and/or updated American College of Rheumatology criteria for the classification of SLE (12) and were consecutively enrolled from January 1, 1999, to December 31, 2009, in 26 centers. The inclusion criteria were: 1) diagnosis of SLE and 2) with ESR measured. The exclusion criteria were: 1) loss to follow-up and 2) not a first hospitalization. The clinical data of 1,217 patients with ESR measured on their first admission were extracted and analyzed for their associations with clinical features, infection status, disease activity, damage accrual, and long-term survival.

The study protocol was reviewed and approved by the Ethics Committee of the Affiliated Drum Tower Hospital of Nanjing University Medical School, and all the data were collected anonymously. All assessments were standardized across centers. Disease activity was evaluated according to the SLE Disease Activity Index (SLEDAI) score (13), and damage accrual was determined using the Systemic Lupus International Collaborating Clinics (SLICC)/American College of Rheumatology (ACR) Damage Index (SDI) (14). Flare was defined as an increase in SLEDAI. The scores and indices were confirmed by two independent clinical rheumatologists. Infection status on admission was decided based on the clinical manifestations, the laboratory tests including microbial detection, the treatment effects, and the discharge diagnosis. All infection cases were adjudicated by one senior rheumatologist and one infectious disease specialist who were blinded to the ESR stratification. Disagreements were resolved via group discussion until consensus was reached.

The ESR measurements were based on a single time-point value within 3 days of SLE admittance. The ESR cutoff values were based on internationally recognized sex-stratified clinical reference ranges—ESR >15 mm/h for men and ESR >20 mm/h for women—which were predefined before data analysis. Normal values for laboratory tests were as follows (10): C-reactive protein (CRP), ≤8 mg/L; serum creatinine, ≤133 μmol/L; serum albumin, ≥35 g/L; serum alanine aminotransferase or aspartate aminotransferase, ≤60 U/L; white blood cells (WBCs), ≥4 × 10^9^/L; red blood cells (RBCs), ≥3.8 × 10^12^/L (women) or 4.3 × 10^12^/L (men); platelets, ≥100 × 10^9^/L; urine protein, <0.5 g/24 h; urine WBC, <9/μl; urine RBC, <5/μl; pathologic cast, <1/μl.

### Patient and public involvement

Patients or the public were not involved in the design or conduct of this study. However, patients participated in the reporting of their survival status and will be informed of the latest findings at the next follow-up.

### Statistical analysis

Statistical analysis was performed using SPSS 24.0 or GraphPad Prism 7.0 software. Enumeration data were expressed as number (percentages), and comparisons between two groups were performed using the chi-square test or the Fisher’s exact test, while measurement data between two groups were analyzed with the unpaired Student’s *t*-test. Multivariate logistic regression analysis was applied to determine clinical features independently associated with ESR (enter method, with variable entry at 0.05). Anemia, hypoalbuminemia, and age were included in the multivariable regression models, no matter the *p*-value of the comparison of the factors between ESR normal and ESR high. Missing data were treated with multiple imputation, and the results were reported as odds ratios (ORs) with 95% confidence intervals (CIs). Pearson’s or Spearman’s rank correlation test was used to analyze the relationship between two variables according to the data distribution. The cutoff values of ESR to judge the disease activity and the CRP/ESR ratio for the differential diagnosis of infection were assessed using receiver operating characteristic (ROC) curves, and the detection capability was evaluated with the area under the ROC curve (AUC). To explore the risk of ESR for mortality in patients with SLE, cumulative survival was illustrated with the Kaplan–Meier plot, and factors were compared using the log-rank test. A *p* < 0.05 was considered statistically significant.

## Results

### Demographics of the patients

Of the patients, 81.0% (986 out of 1,217) had increased ESR values, 81.7% of whom were less than 45 years old and the remaining 18.9% over 45 years old. Women comprised 92.3%, and the median disease duration was 1.1 years. Of the patients, 86.8% had SLEDAI scores ≥7 and 12.4% had SDI scores ≥1 on admission. Organ involvement was relatively high in these patients, and the features most often observed were mucocutaneous (66.4%), musculoskeletal (55.0%), renal (51.8%), and hematologic (45.4%) involvements, while cardiopulmonary (21.4%), gastrointestinal (20.0%), and neuropsychiatric (6.6%) involvements were less common.

### Association of ESR with clinical features

**Figure 1:**
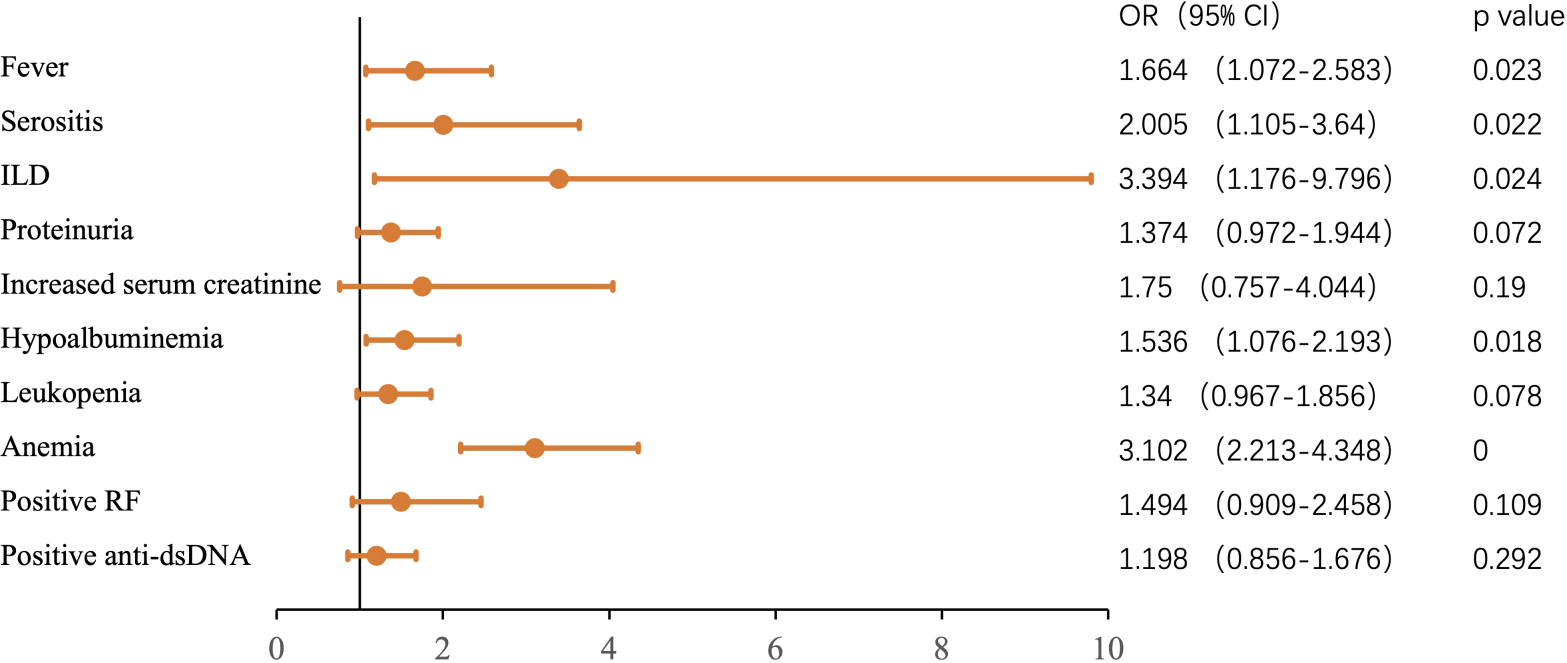
Multivariate-adjusted odds ratios (95% confidence intervals) of various clinical features for erythrocyte sedimentation rate (ESR) elevation in systemic lupus erythematosus (SLE) patients.

The ESR value was not associated with age, gender, or disease duration. More patients with high ESR displayed fever, serositis, interstitial pneumonia, proteinuria, increased serum creatinine, hypoalbuminemia, leukopenia, and anemia (all *p* < 0.05) (**Table 1**). Interestingly, there was an association between high ESR and positive rheumatoid factor (RF) or positive anti-dsDNA (*p* < 0.0001 and *p* = 0.0001, respectively). We then performed multivariate logistic regression analysis to verify the link between the different variables and a high ESR. As shown in **Figure 1**, ESR elevation was independently associated with fever (OR = 1.664, 95%CI = 1.072–2.583), serositis (OR = 2.005, 95%CI = 1.105–3.64), interstitial pneumonia (OR = 3.394, 95%CI = 1.176–9.796), hypoalbuminemia (OR = 1.536, 95%CI = 1.076–2.193), and anemia (OR = 3.102, 95%CI = 2.213–4.348). There was no difference between the ESR normal and ESR high groups according to the use of glucocorticoid or hydroxychloroquine. A high ESR may have an association with the use of immunosuppressants (*p* < 0.01). However, no difference was found when specific immunosuppressants were compared.

**Table 1:** Associations of the clinical features with the erythrocyte sedimentation rate (ESR) in 1,217 systemic lupus erythematosus (SLE) patients.

Variable	ESR normal (*n* = 231)	ESR high (*n* = 986)	*P*-value
Gender (female)	214 (92.6%)	909 (92.2%)	>0.05
Age ≤45 years	190 (82.3%)	804 (81.5%)	>0.05
Age >45 years	41 (17.7%)	182 (18.5%)	>0.05
Time to diagnosis (≥2 years)	47 (23.0%)	207 (23.1%)	>0.05
Disease duration (≥3 years)	87 (38.0%)	332 (33.8%)	>0.05
Fever	28 (12.3%)	236 (24.2%)	<0.001
Mucocutaneous involvement			
Rash	127 (55.0%)	590 (59.8%)	>0.05
Mucosal ulcer	29 (12.6%)	97 (9.8%)	>0.05
Cutaneous vasculitis	17 (7.4%)	101 (10.2%)	>0.05
Alopecia	27 (11.7%)	106 (10.8%)	>0.05
Neuropsychiatric involvement			
CNS manifestations	10 (4.3%)	63 (6.4%)	>0.05
Cardiopulmonary involvement			
Myocarditis	2 (0.9%)	11 (1.1%)	>0.05
Serositis	14 (6.1%)	180 (18.3%)	<0.001
Interstitial pneumonia	4 (1.7%)	61 (6.2%)	<0.01
Musculoskeletal involvement			
Myositis	6 (2.6%)	27 (2.7%)	>0.05
Arthritis	117 (50.6%)	537 (54.5%)	>0.05
Renal involvement			
Hypertension	21 (9.1%)	111 (11.3%)	>0.05
Proteinuria	78 (33.8%)	494 (50.1%)	<0.001
Increased serum creatinine	9 (4.2%)	83 (9.0%)	<0.05
Active urinary sediment	6 (2.6%)	33 (3.3%)	>0.05
Gastrointestinal involvement			
Serum transaminase increase	35 (16.1%)	156 (16.6%)	>0.05
Hypoalbuminemia	80 (37.0%)	584 (63.5%)	<0.001
Hematologic involvement			
Leukopenia	42 (18.2%)	283 (28.7%)	0.001
Thrombocytopenia	60 (26.0%)	271 (27.5%)	>0.05
Anemia	46 (20.0%)	480 (49.8%)	<0.001
Immunology features			
Positive rheumatoid factor	22 (15.2%)	201 (31.3%)	<0.001
Positive anti-dsDNA	86 (41.7%)	495 (54.7%)	0.001
Positive anti-Sm	58 (28.7%)	311 (34.9%)	>0.05
Low complement C3	147 (70.7%)	651 (74.9%)	>0.05
Medication			
Steroids	219 (94.8%)	911 (92.4%)	>0.05
Anti-malarial drugs	99 (42.9%)	409 (41.5%)	>0.05
Immunosuppressives	108 (46.8%)	555 (56.3%)	<0.01
Cyclophosphamide	91 (39.4%)	434 (44.0%)	>0.05
Mycophenolate mofetil	5 (2.2%)	32 (3.2%)	>0.05
Azathioprine	2 (0.2%)	21 (2.1%)	>0.05
Methotrexate	7 (3.0%)	48 (4.9%)	>0.05
Calcineurin inhibitors	4 (1.7%)	6 (0.6%)	>0.05
Leflunomide	2 (0.9%)	29 (2.9%)	>0.05
*Tripterygium wilfordii* multiglycosides	7 (3.0%)	49 (5.0%)	>0.05

Values shown are number (percentages). For the majority of variables, the values presented may be lower than the overall numbers due to incomplete data collection.

*CNS*, central nervous system.

### Association of ESR with disease activity

**Figure 2:**
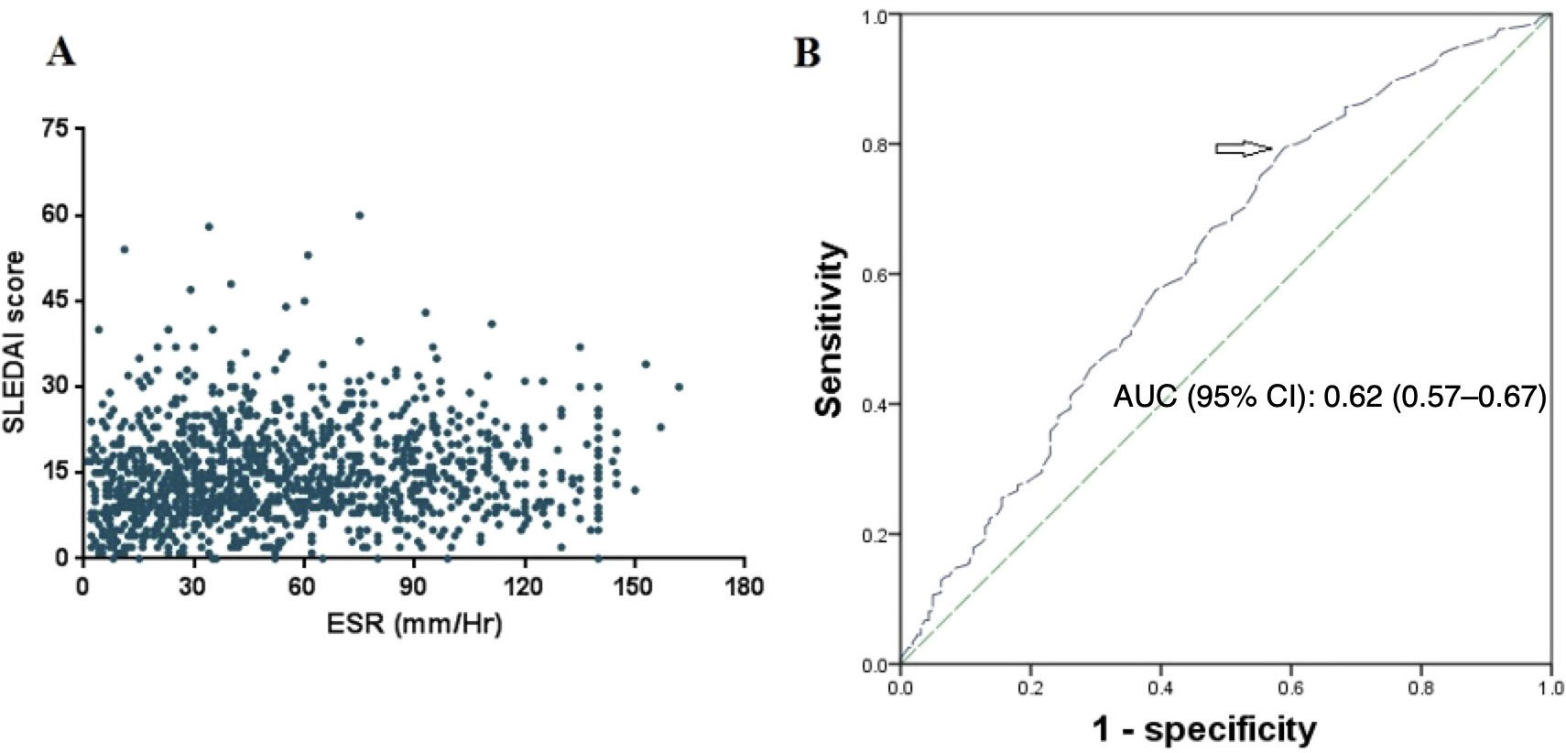
Association of erythrocyte sedimentation rate (ESR) with systemic lupus erythematosus (SLE) disease activity. **(A)** Distribution of the ESR values among different SLE Disease Activity Index (SLEDAI) scores. **(B)** Receiver operating characteristic (ROC) curve for ESR for the detection of active disease (SLEDAI score ≥7). *Arrow* denotes optimal cutoff.

The ESR values of patients with SLE were correlated with their SLEDAI scores (Spearman’s *r* = 0.15, *p* < 0.0001) (**Figure 2A**). In contrast, there was only a weak association between the ESR values and the SDI scores (Spearman’s *r* = 0.06, *p* < 0.05). Using ROC analysis, we then evaluated whether the ESR values would allow discriminating patients with moderate and severe disease activities (defined as SLEDAI score ≥7) from those without. As shown in **Figure 2B**, the AUC was 0.62 (95%CI = 0.57–0.67, *p* < 0.01). Corresponding to the point on the ROC curve where the Youden index (sensitivity + specificity −1) is the highest, the optimal cutoff value of ESR for detecting lupus activity was 24.50 mm/h (with sensitivity and specificity of 79.5% and 41.0%, respectively).

### Association of ESR with infection

**Figure 3:**
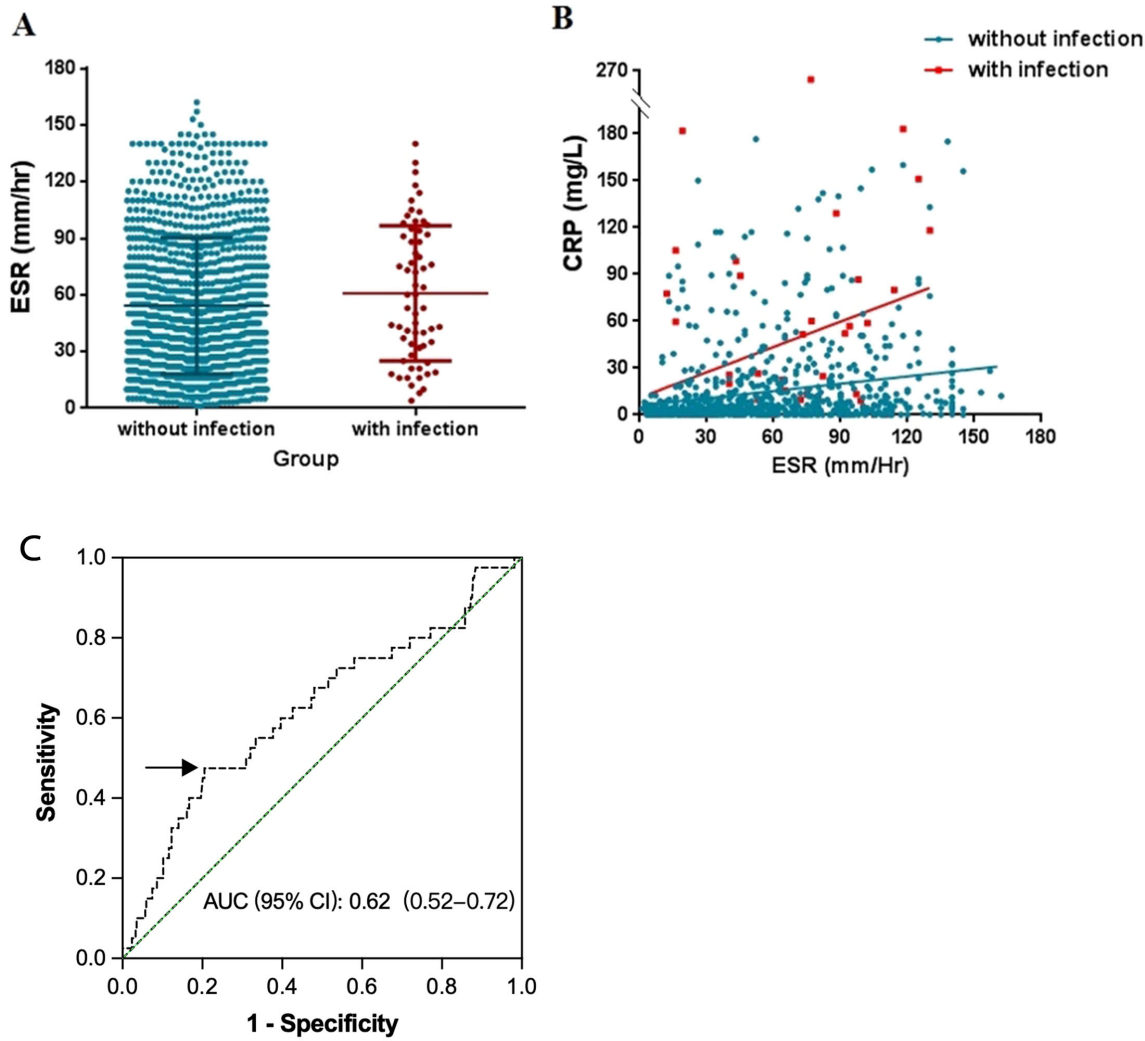
Association of erythrocyte sedimentation rate (ESR) with infection. **(A)** ESR values in systemic lupus erythematosus (SLE) patients with or without concurrent infection. **(B)** Trends in the association between C-reactive protein (CRP) and ESR in patients with or without infection. **(C)** Receiver operating characteristic (ROC) curve for the CRP/ESR ratio for differential diagnosis of infection. *Arrow* denotes optimal cutoff.

Among 1,217 patients, 62 (5.1%) were identified to have an infection at the time of admission. The ESR level was closely associated with the CRP value (Spearman’s *r* = 0.28, *p* < 0.0001), but was not elevated in patients with concurrent infection (**Figure 3A**). With the ESR unchanged, the increase of CRP in patients with infection was three to four times higher than those without (**Figure 3B**). For patients with elevated ESR levels, a CRP/ESR ratio ≥0.5 helped distinguish infections (**Figure 3C**). The 0.5 threshold was determined using ROC analysis, in which the sensitivity, specificity, positive predictive value (PPV), negative predictive value (NPV), and AUC were 47.5%, 79.5%, 11.07%, 96.57%, and 0.62 (95%CI = 0.52–0.72, *p* < 0.01), respectively. The cutoff value corresponded to the point on the ROC curve with the highest Youden index. In summary, the rates of the different types of infections were as follows: pulmonary infection (26/62, 41.9%) and upper respiratory tract infection (14/62, 22.6%) were the most frequent. There were fewer numbers of other types of infections, as follows: three urinary tract infections, three herpes zoster infections, two central nervous system infections, one acute gastroenteritis, one tuberculosis, one skin infection, one sepsis, one oropharyngeal fungal infection, and nine cases of unknown cause (not recorded).

### ESR and patient outcomes

**Figure 4:**
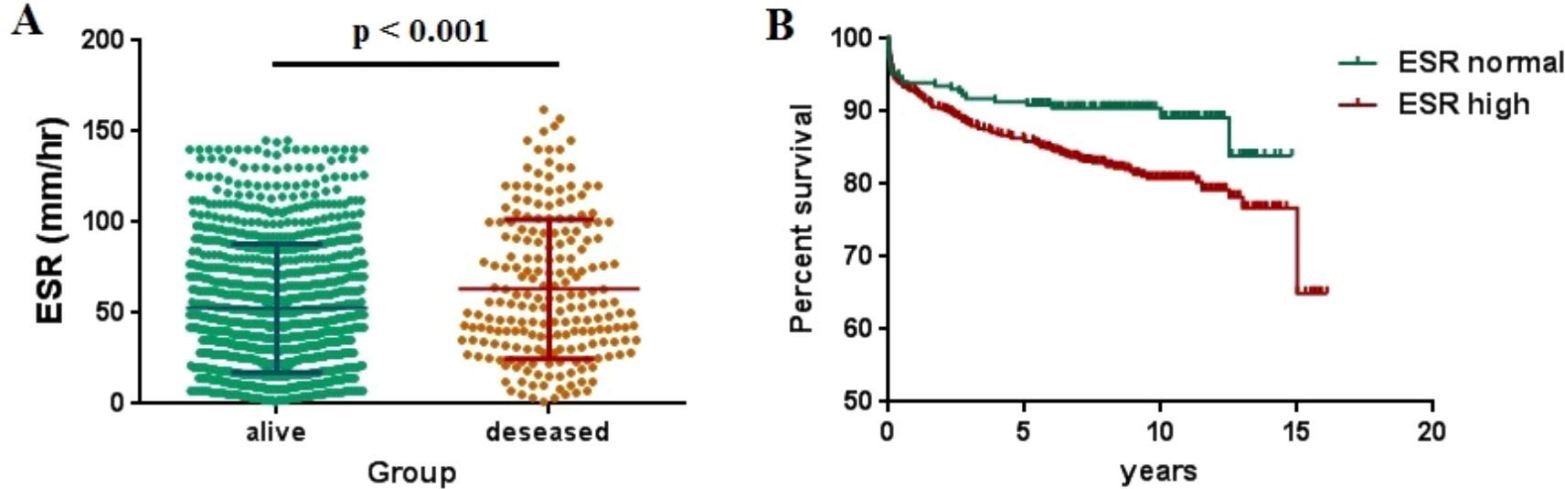
Association of erythrocyte sedimentation rate (ESR) with long-term outcome. **(A)** ESR values at first admission in surviving or deceased patients. **(B)** Kaplan–Meier survival estimates according to the ESR values.

In this cohort, 205 patients (16.8%) died during the follow-up. The patients who died had higher ESR values than those who are still alive (*p* < 0.001) (**Figure 4A**). Consistently, those with elevated ESR had poor outcomes according to the Kaplan–Meier survival analysis. The 5- and 10-year survival rates for patients with high ESR values were 85.9% and 80.8%, respectively, which were significantly lower than those with normal ESR values (90.9% and 89.1%, respectively, *p* < 0.01) (**Figure 4B**).

## Discussion

ESR is one of the most commonly used markers of inflammation in clinical practice. However, its role in the course of SLE disease remains unclear. Recently, it has even been argued that ordering an ESR test may often be unnecessary due to the change in the globulin gap always reflecting changes in the ESR among patients with a high globulin gap (15). In this study, by analyzing the data of 1,217 patients with SLE, originally from 26 centers across Jiangsu Province, China, it was demonstrated that, other than anemia and hypoalbuminemia, ESR was also independently associated with fever, serositis, and interstitial pneumonia. An ESR value over 25 mm/h might indicate lupus activity with high sensitivity. On the other hand, the CRP/ESR ratio helped determine the existence of infection with high specificity. Patients with elevated ESR had lower long-term survival rates and thus needed prompt treatment.

A major problem in the application of ESR in clinical assessment is that this indicator is related to a lot of factors, such as female gender, older age, anemia, hypoalbuminemia, and pregnancy. In SLE, an elevation in ESR is often attributed to changes in the serum proteins or erythrocytes (4), while age and gender appear less important as ESR correction has little impact on the interpretation of the results (16). Recently, a high ESR has been suggested as a risk factor for serositis, in particular pericarditis, in another Chinese cohort (17). Consistently, our data confirmed a link between ESR and anemia, hypoalbuminemia, and serositis. Moreover, we showed for the first time that ESR elevation is associated with interstitial pneumonia in patients with SLE, suggesting that a patient’s lung should be checked when the ESR value is high.

Differently from CRP, ESR is not a sensitive indicator of infection (5, 18, 19). However, due to their different rates of increase at the time of infection, the combination of ESR and CRP may improve the diagnostic sensitivity. Recently, in an observation of 53 hospitalizations, the ratio of ESR to CRP has been shown to be useful in distinguishing an infection from a flare in patients with SLE presenting with fever (20). In this study, we demonstrated that, for patients with a higher than normal ESR, a CRP/ESR ratio over 0.5 may suggest the presence of infection whether or not they had a fever or a disease flare. An NPV of 96.57%, which is for the cutoff CRP/ESR in this study, may imply a potential role in clinical use. In other studies mentioned in a systematic review, the use of CRP or ESR alone was not satisfactory enough (21). Comparisons or combinations with additional biomarkers such as procalcitonin would enhance clinical applicability (22). However, these data were not systematically available in our cohort. Leukocyte indices such as the neutrophil-to-lymphocyte ratio (NLR) and the systemic immune-inflammation index (SII), among others, are novel biomarkers for the evaluation of SLE disease activity and infection (23, 24). We shall be comparing these biomarkers with the CRP/ESR ratio or combining these indices in different scenarios in the future. Survival analysis showed that an elevated ESR is associated with poorer 5- and 10-year survival rates. However, based on the existing confounding factors, an elevated ESR may reflect the overall inflammatory burden rather than a purely independent prognostic factor. Moreover, due to the ESR essentially representing RBC aggregation and plasma resistance, more molecules and mechanisms linking changes in the ESR and the status of autoimmune diseases need to be explored in the future.

As this is a cross-sectional study, the data should be interpreted within the context of several limitations. Firstly, the SLE patients in this cohort were not randomly selected and only those hospitalized were included. Restricting the cohort to hospitalized patients may have introduced selection bias toward patients with more severe disease. Similarly, patients might have suffered more severe infection, needing in-hospital intravenous drip of antibiotics. As hospitalized patients have more thorough information, including comprehensive laboratory results and SLEDAI evaluation, a large sample size and comprehensive multivariate analyses may help decrease, although not eliminate, this potential bias. Secondly, despite efforts to maximize data collection, a small number of records were still missing, which might have caused statistical bias. Thirdly, we did not track changes in the ESR or CRP over time. Longitudinal studies are needed to evaluate dynamic changes in ESR over time. It is also extremely important to emphasize that the cutoff values—25 mm/h for ESR and ≥0.5 for the CRP/ESR ratio—were obtained using ROC curve analysis based on this current dataset. Although these cutoff values showed good diagnostic performance in our cohort, potential overfitting may exist due to the lack of external validation. Further multicenter studies are needed to verify these findings. This cohort only included a Chinese population, with a single ethnic composition, which limits generalization of the research results and cannot be directly extended to SLE patients of other races/from other regions. Potential residual confounding still exists, such as medication changes and socioeconomic status, which were not recorded and may have influenced the results.

In conclusion, in this study, we analyzed and summarized changes in the ESR in a large group of Chinese patients with SLE. The results may be used as a reference for clinical practice. In this cohort, ESR showed correlations with fever, serositis, interstitial pneumonia, hypoalbuminemia, and anemia. ESR higher than 25 mm/h might discriminate patients with moderate and severe disease activities. Furthermore, the CRP/ESR ratio (≥0.5) might indicate infection in patients with SLE. Ultimately, an elevated ESR is associated with poorer 5 and 10-year survival.

## Data Availability

The raw data supporting the conclusions of this article will be made available by the authors, without undue reservation.
