# Correction: Clinical significance of erythrocyte sedimentation rate-based stratification in a large retrospective SLE cohort

**DOI:** 10.3389/fimmu.2026.1881509

**Published:** 2026-05-22

**Authors:** Fan Wang, Yang Xu, Wei Zhang, Wenyou Pan, Lin Liu, Min Wu, Fuwan Ding, Huaixia Hu, Xiang Ding, Hua Wei, Yaohong Zou, Wei Kong, Yun Zhu, Xuebing Feng, Lingyun Sun

**Affiliations:** 1 Department of Rheumatology and Immunology, Nanjing Drum Tower Hospital, Affiliated Hospital of Medical School, Nanjing University, Nanjing, China; 2 Department of Rheumatology and Immunology, Nanjing Medical University Drum Tower Clinical Medical Hospital, Nanjing, China; 3 Department of Rheumatology, Huaian First People’s Hospital, Huaian, China; 4 Department of Rheumatology, Xuzhou Central Hospital, Xuzhou, China; 5 Department of Rheumatology, The Third Affiliated Hospital of Soochow University, Changzhou, China; 6 Department of Endocrinology, Yancheng Third People’s Hospital, Yancheng, China; 7 Department of Rheumatology, Lianyungang Second People’s Hospital, Lianyungang, China; 8 Department of Rheumatology, Lianyungang First People’s Hospital, Lianyungang, China; 9 Department of Rheumatology, Northern Jiangsu People’s Hospital, Yangzhou, China; 10 Department of Rheumatology, Wuxi People’s Hospital, Wuxi, China

**Keywords:** systemic lupus erythematosus, erythrocyte sedimentation rate, C-reactive protein, disease activity, infection

Affiliation “Department of Rheumatology and Immunology, Nanjing Drum Tower Hospital, Affiliated Hospital of Medical School, Nanjing University, Nanjing, China” was erroneously given as “Department of Rheumatology and Immunology, The Affiliated Drum Tower Hospital of Nanjing University Medical School, Nanjing, China”.

The original version of this article has been updated.

